# Neuronal apoptosis inhibitory protein is implicated in amyotrophic lateral sclerosis symptoms

**DOI:** 10.1038/s41598-017-18627-w

**Published:** 2018-01-08

**Authors:** Osamu Kano, Kazunori Tanaka, Takuya Kanno, Yasuo Iwasaki, Joh-E Ikeda

**Affiliations:** 1 Division of Neurology, Department of Internal Medicine, School of Medicine, Faculty of Medicine, Toho University, Tokyo, 143-8541 Japan; 2 NGP Biomedical Research Institute, Neugen Pharma Inc., Tokyo, 153-0051 Japan; 3 CMIC Pharma Science Co., Ltd., Hokuto, Yamanashi 408-0044 Japan; 4 Department of Molecular Neurology, Kitasato University Graduate School of Medical Sciences, Sagamihara, Kanagawa 252-0374 Japan; 5 Apoptosis Research Centre, Children’s Hospital of Eastern Ontario, Ottawa, Ontario, K1H 8L1 Canada; 6 Department of Pediatrics, Faculty of Medicine, University of Ottawa, Ottawa, Ontario, K1H 8M5 Canada; 7 Department of Molecular Life Sciences, Tokai University School of Medicine, Isehara, Kanagawa 259-1193 Japan

## Abstract

The delineation of the molecular pathology underlying amyotrophic lateral sclerosis (ALS) is being hampered by the lack of suitable biomarkers. We have previously reported that bromocriptine upregulates the endogenous antioxidative factor, neuronal apoptosis inhibitory protein (NAIP), sustains motor function and slows disease progression in ALS patients, implying the NAIP’s implication in ALS. Here, we aimed to verify a correlation of NAIP level with disease progression in ALS patients. The amount of NAIP in mononuclear cells (MNC) from peripheral blood from ALS patients (n = 18) and the age matched healthy controls (n = 12) was validated by NAIP-Dot blotting. Notably, the MNC-NAIP level in ALS patients (0.62 ± 0.29 ng) was nearly half of that in the healthy controls (1.34 ± 0.61 ng, P = 0.0019). Furthermore, the MNC-NAIP level in ALS patients and their ALS Functional Rating Scale-Revised (ALSFRS-R) score were evaluated through 1 year. Regression analysis of the MNC-NAIP vs ALSFRS-R indicated that a higher amount of MNC-NAIP was associated with a smaller change in ALSFRS-R at 12 months (R^2^ = 0.799; P = 0.016), suggesting that a progressive increment of the MNC-NAIP led to slower ALS progression. Our present report implies that NAIP will have broad implications for ALS symptoms as a risk factor and a promising prognostic biomarker.

## Introduction

Amyotrophic lateral sclerosis (ALS) is an adult-onset neurodegenerative disorder characterized by rapidly progressive paralysis and death due to respiratory failure, typically within 2–3 years of disease onset^1,2^. Approximately 10% of ALS are familial, whereas the remaining are sporadic. Although several causative genes for ALS have been identified thus far, and genetic etiology is known to be responsible in two-thirds of familial cases and in approximately 11% of sporadic ALS cases^3^, the etiology in the other cases remains unclear. A complex interplay of many pathogenic factors, including oxidative stress, excitotoxicity, mitochondrial dysfunction, disruption of the neurofilament network, neuronal inflammation, non-cell autonomous damage, and protein aggregation (such as SOD1, TARDBP, C9ORF72, and FUS) have been suggested as potential factors^4–8^. Among these, there is substantial evidence to support the hypothesis that oxidative stress and chronic neuronal inflammation play crucial roles in ALS pathogenesis^9^, which consequently led to novel insights in the development of effective treatments in ALS.

Riluzole—an antiglutamatergic agent—has been approved by the Food and Drug Administration (FDA) for the treatment of ALS^10,11^, and edaravone—a free radical scavenger—was recently approved by the Pharmaceutical and Medical Devices Agency (Japan) in 2015 and by the FDA in 2017 for the treatment of ALS^12^. Nevertheless, these medications only have a modest impact. More than 50 randomized controlled trials (RCTs) of proposed disease-modifying drugs have failed to show positive results in the past half-century^13^. In fact, in the last decade alone, at least 18 drugs have been tested in large phase 2 or 3 RCTs. The most obvious deficiency in these trials is the lack of objective biomarkers. Despite the currently available knowledge on ALS, no method for testing the pathogenic targets in patients is available. RCTs have evaluated the efficacy of drugs in terms of the clinical effect based on the ALS Functional Rating Scale-Revised (ALSFRS-R) score. Hence, the present clinical trials cannot distinguish those ALS patients who responded or did not respond to the drug due to the lack of an ALS biomarker, which may have resulted in an ambiguous evaluation. Thus, the identification of a simple biomarker and a biomarker-based clinical trial in ALS may offer a solution to this impregnable dilemma.

Neuronal apoptosis inhibitory protein (NAIP), which is thought to be a modifier gene for spinal muscular atrophy, is a founding member of the inhibitor of apoptosis^14^. NAIP has also been classified as a nucleotide binding-oligomerization domain and leucine-rich repeat (NLR) protein^15^. Recent findings have reported that NAIP plays a crucial role in the host defense against bacterial infection^16,17^, and also forms a multiprotein complex with NLRC4 (NLR family, CARD domain containing 4) to induce inflammasome activation via bacterial infection^18–22^. Importantly, NAIP selectively suppresses the cell death induced by oxidative stress. In fact, studies have shown that upregulated and overexpressed NAIP protects neuronal cells from oxidative injuries *in vitro* and *in vivo* ^23–25^. Hence, NAIP is thought to be a multifunctional protein.

Given the action of NAIP against oxidative stress, we previously established a NAIP-ELISA-based drug screening system for the development of novel ALS drugs. Accordingly, we identified the NAIP-upregulated small molecule compound L-745,870 as a drug candidate for antipsychotic treatment, bromocriptine mesylate (BRC) as a Parkinson’s disease drug, and WN1316 as a drug candidate for ALS^24–27^. Indeed, the post-onset administration of these compounds alleviated disease progression in an ALS (SOD1^H46R^) and (SOD1^G93A^) mouse models^26,28^. Interestingly, clinical research showed that the motoneuronal function in ALS patients was sustained in part by the BRC treatment^29^. As BRC is an NAIP-upregulating compound, we hypothesized that the NAIP level might be low in ALS patients, in comparison with that in healthy controls, and may recover in ALS patients to a certain extent following BRC treatment.

In this study, we assessed whether the NAIP levels change in ALS patients. Most tissues and cells (except for hematopoietic tissues) express NAIP at very low levels^27^. In the present study, NAIP expression was noted in human peripheral hematopoietic cells, particularly in mononuclear cells, but not in erythrocytes and polymorphonuclear leukocytes. Hence, to measure the amount of NAIP in both ALS patients and healthy controls, we performed dot blot analysis with anti-NAIP antiserum using whole protein extract of mononuclear cells (MNC) from peripheral blood. We further longitudinally evaluated the relationship between the MNC-NAIP level and ALSFRS-R up to 12 months as an indicator of disease progression. We found that the MNC-NAIP level was markedly low in ALS patients less than half amount of the healthy control, suggesting that NAIP may serve as a risk factor for ALS. Furthermore, we found that ALS patients who exhibited longitudinal increases in the MNC-NAIP level retained motoneuronal function to some extent up to 12 months testing period, but no such retention was observed in ALS patients with rapid disease progression. Thus, our study strongly suggests that MNC-NAIP may serve as an easily accessible prognostic biomarker.

## Results

### Baseline characteristics of ALS patients and healthy controls

In the present study, we aimed to assess the change in MNC-NAIP, ALSFRS-R, and %FVC in 18 sporadic ALS patients during the 12 months testing period. Among them, 6 patients were followed up to 12 months. On the other hands, 3 patients died within testing period, 3 could not visit our clinic at fixed testing intervals, and 6 discontinued after participating in edaravone clinical study. Therefore, the rest of 12 ALS patients were followed for <12 months.

The populations of ALS patients and healthy controls in the present study were similar in terms of age at baseline (64.1 years vs. 59.8 years), but different in gender (Table 1). The clinical characteristics of each ALS patient, including ALSFRS-R; %FVC; and riluzole, PEG, and NIV use, are summarized in Supplementary Table S1.

**Table 1:** Summary of the demographic and baseline characteristics.

	ALS (n = 18)	Control (n = 12)
age, year (SD)	64.1 (12.3)	59.8 (11.9)
male/female, n	13/5	4/8
clinical onset, n (%)		
bulbar	6 (33.3)	
upper limb	9 (50.0)	
lower limb	3 (16.7)	
duration months (SD)	27.6 (16.3)	
ALSFRS-R score	37.1 (7.4)	
forced vital capacity, % (SD)	77.3 (27.5)	
riluzole, n (%)	18 (100)	
PEG, n (%)	11 (61.1)	
NIV, n (%)	7 (38.9)	

ALSFRS-R: Amyotrophic Lateral Sclerosis Functional Rating Scale-Revised, PEG: percutaneous endoscopic gastrostomy, NIV: noninvasive ventilation, n: number, SD: standard deviation.

### MNC-NAIP levels in ALS patients are lower than those of healthy controls at baseline

First, to analyze the NAIP expression in MNC, polymorphonuclear cells, and erythrocytes, we performed Western blotting with anti-NAIP antiserum. In addition, we detected specific MNC-NAIP expression in peripheral blood (Supplementary Fig. S1).

**Figure 1:**
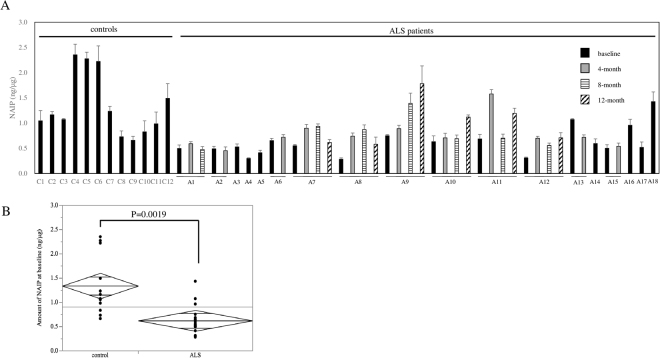
Comparison of the amount of MNC-NAIP between healthy controls and ALS patients. (**A**) Dot blot analysis of NAIP using mononuclear cells (MNC) from healthy controls and ALS patients was performed with anti-NAIP antiserum. The density of each NAIP dot signal was quantified per μg of whole protein extract from the MNC. The black, gray, horizontal lines and slash line bars indicate the amount of NAIP at baseline, 4 months, 8 months, and 12 months, respectively. Data are expressed as mean ± SD (n = 4 technical replicates). Case A7–A12 were followed up to 12 months. Case A2, A4, and A14 died within testing period. Case A1, A5, and A6 could not visit clinic at fixed testing intervals. Case A3, A13, A15–A18 discontinued after participating in edaravone clinical study. (**B**) The amount of MNC-NAIP at baseline between ALS patients (n = 18) and healthy controls (n = 12) is shown as Mean Diamonds. P = 0.0019 by t test.

Next, to evaluate the amount of MNC-NAIP at baseline, we conducted dot blotting and quantitated per μg of whole protein extract of MNC from 18 ALS patients and 12 healthy controls (n = 4 technical replicates; Fig. 1A). The MNC-NAIP levels in healthy controls and in ALS patients ranged from 0.66 to 2.35 ng/μg (average, 1.34 ± 0.61 ng/μg) and from 0.28 to 1.43 ng/μg (average, 0.62 ± 0.29 ng/μg), respectively (Fig. 1B). We have also statistically analyzed the data excepting 3 healthy control high outliers and NAIP levels in ALS patients still significantly decreased compared with the healthy controls (P = 0.0018). When we compared the gender difference in each group, the average MNC-NAIP levels in male (n = 13) and female (n = 5) ALS patients were 0.71 ± 0.29 ng/μg and 0.40 ± 0.14 ng/μg, respectively. In that of healthy male (n = 4) and female (n = 8) controls were 1.77 ± 0.66 ng/μg and 1.12 ± 0.49 ng/μg, respectively. Some gender difference was observed in ALS patients (P = 0.04), but the difference between ALS patients and healthy control male and female groups were P = 0.04 in male, and P < 0.01 in female. Hence, there was no correlation between the MNC-NAIP level and age, clinical onset, disease duration, PEG/NIV use in both the healthy controls and ALS patients (Supplementary Table S1). These results suggest that the MNC-NAIP levels were markedly lower in ALS patients than in healthy controls. Moreover, we examined the MNC-NAIP levels in Japanese with Parkinson’s disease, and the NAIP level in MNC from Parkinson’s disease patients was as high as that of healthy controls (average, 0.91 ± 0.46 ng/μg). 8 of 11 patients were being treated with the dopamine agonist (as shown in Supplementary Table S1), therefore the effect of polypharmacy cannot be excluded.

### Longitudinal ALS progression and change in the MNC-NAIP level

**Figure 2:**
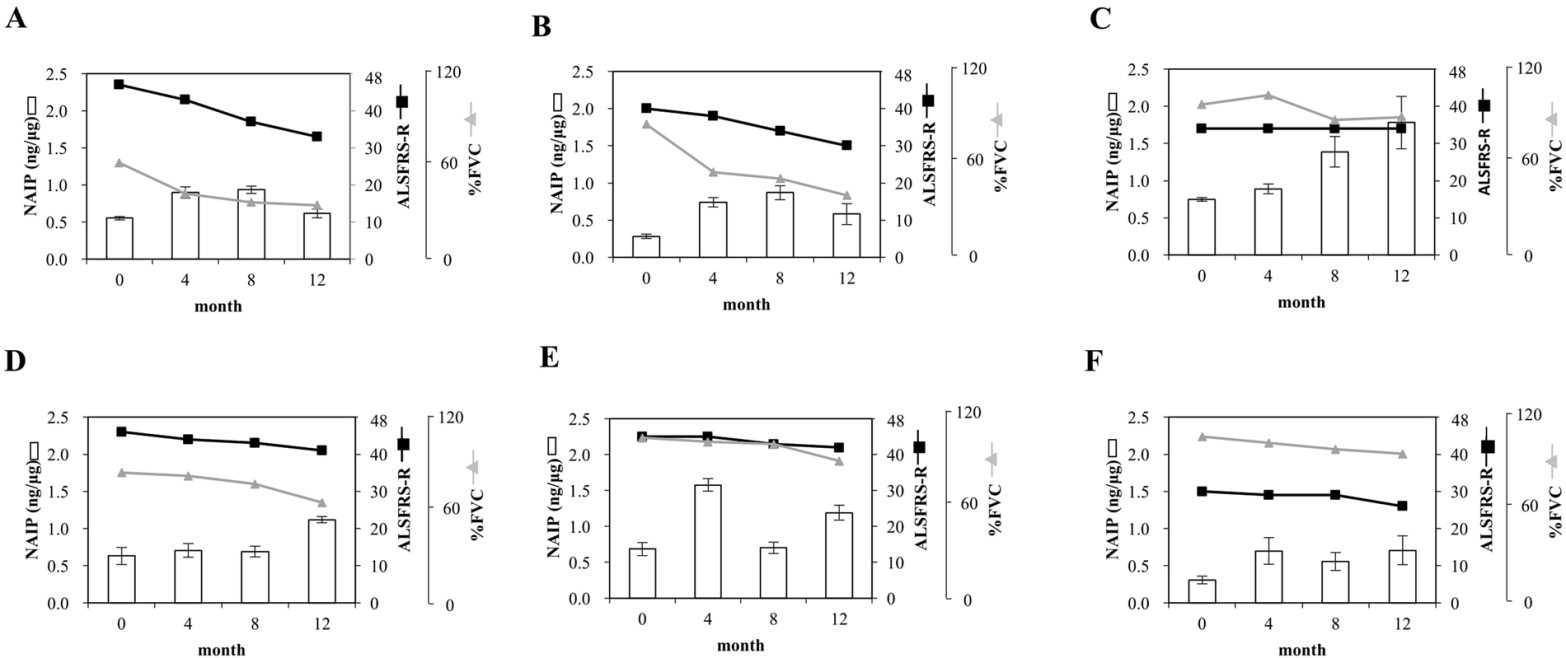
Longitudinal changes in the amount of MNC-NAIP, ALSFRS-R, and %FVC in 6 ALS patients. (**A**–**F**) The NAIP level at baseline, 4 months, 8 months, and 12 months from 6 ALS patients measured using dot blotting was quantitated per μg of whole protein extract from MNC (white bar). The ALSFRS-R and %FVC at baseline, 4 months, 8 months, and 12 months from 6 ALS patients are shown as black squares and gray triangles, respectively. Data are expressed as mean ± SD (n = 4 technical replicates). The information of each ALS patient is follows: (**A**) A 58-year-old man with upper limb type ALS and a disease duration of 16 months (A7); (**B**) A 71-year-old woman with bulbar type ALS and a disease duration of 18 months (A8); (**C**) A 58-year-old man with upper limb type ALS and a disease duration of 77 months (A9); (**D**) A 30-year-old man with lower limb type ALS and a disease duration of 16 months (A10); (**E**) A 65-year-old man with upper limb type ALS and a disease duration of 23 months (A11); (**F**) A 44-year-old woman with upper limb type ALS and a disease duration of 38 months (A12).

To determine whether the amount of MNC-NAIP was correlated with ALS progression, we longitudinally evaluated the NAIP level in MNC from ALS patients by dot blotting and assessed the correlation between the MNC-NAIP levels and the ALSFRS-R/%FVC ratio. In case A9, A10, A11, and A12, an increment of the MNC-NAIP level was observed between baseline and 12 months (Figs 1A and 2C–F, Supplemenatry Table S1), and the amount of MNC-NAIP at 12 months ranged from 0.71 to 1.78 ng/μg (the MNC-NAIP level at 12 months was 1.8 to 2.4-fold greater than that at baseline level; Supplementary Table S1). The longitudinal increase of MNC-NAIP showed no significant change or slight reduction of the ALSFRS-R score and %FVC in these 4 ALS patients (Fig. 2C–F). Interestingly, a significant increase in MNC-NAIP revealed no change of ALSFRS-R score and % FVC (Fig. 2C).

Although the MNC-NAIP level was found to have increased at 12 months (0.58 ng/μg; a 2.1-fold increase as compared to the baseline level; Supplementary Table S1) in case A8 (who showed bulbar type at clinical onset), the ALSFRS-R score and %FVC had reduced during the 12 months testing period (Fig. 2B). Furthermore, in 6 ALS patients, including case A7 (testing period up to 12 months), case A1 (testing period up to 8 months), and cases A2, A6, A13, and A15 (testing period up to 4 months), the MNC-NAIP level showed almost no change or indicated a slight decrease (0.45–0.72 ng/μg; less than 1.1-fold) as compared to the baseline level (Supplementary Table S1). The steady or slight decrease in MNC-NAIP was accompanied by a marked decrease of ALSFRS-R score and %FVC (Fig. 2A,B and Supplementary Table S1). These results imply that an increment of MNC-NAIP to a critical amount is capable to reduce ALS symptoms.

### Regression analysis between the change in MNC-NAIP levels and ALSFRS-R

**Figure 3:**
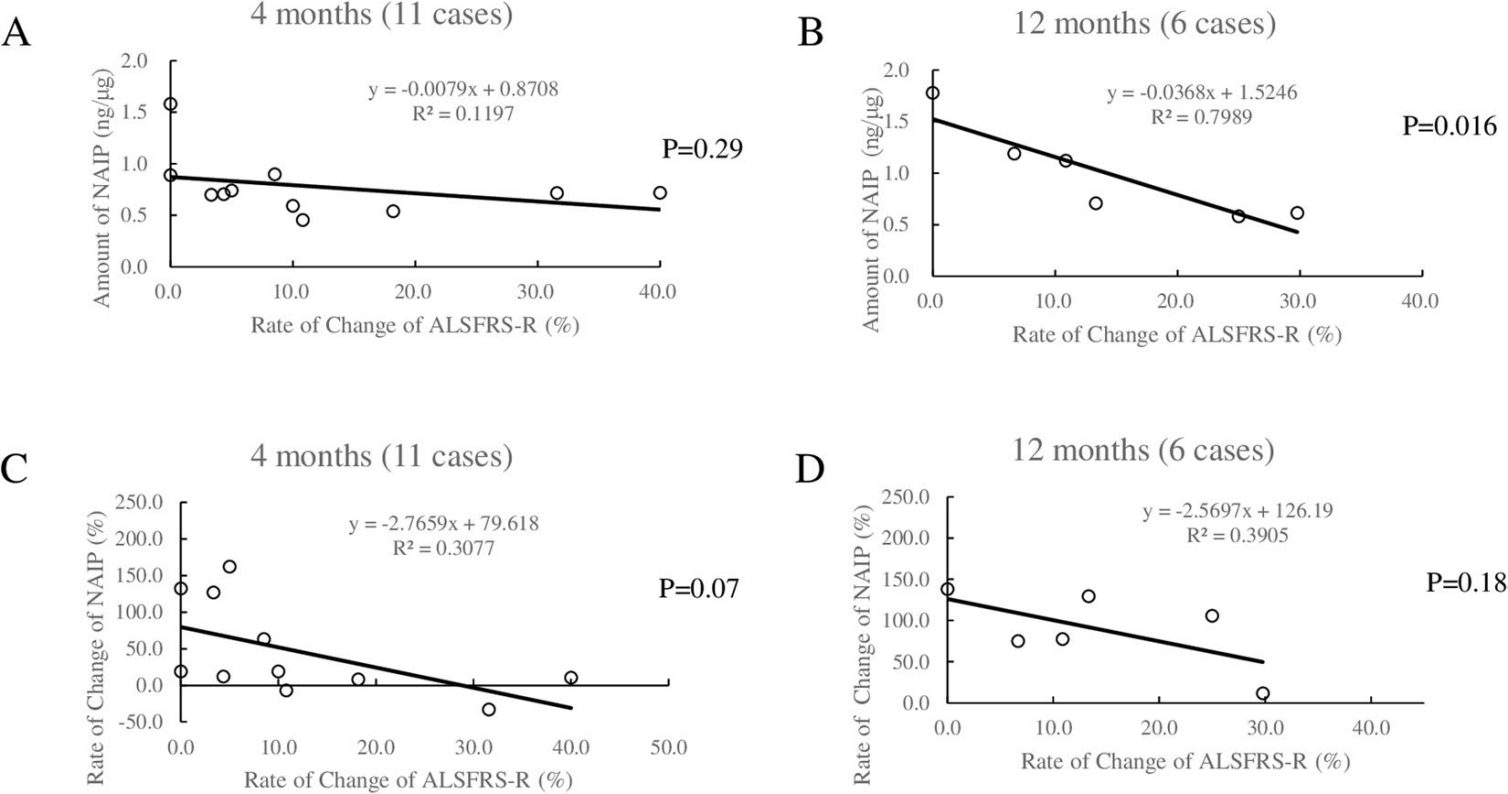
Regression analysis of MNC-NAIP levels and ALSFR-R. Eleven ALS patients at 4 months (case A1, A2, A6–A13, and A15) and 6 ALS patients at 12 months (Case A7–A12) were chosen for the evaluation of regression analysis. Correlations between the amount of MNC-NAIP and the rate of change in ALSFRS-R at 4 months (**A**) and 12 months (**B**) and between the rate of change in the amount of MNC-NAIP and the rate of change in ALSFRS-R at 4 months (**C**) and 12 months (**D**) are shown.

To further verify the correlation between the amount of NAIP and the rate of change in the ALSFRS-R score, we performed a detailed examination of the regression statistics of the results (Supplementary Table S1). We observed a significant correlation between the amount of MNC-NAIP and the rate of change in the ALSFRS-R at 12 months (P = 0.016; R^2^ = 0.798; Fig. 3B). Moreover, the rate of change of the amount of MNC-NAIP was also correlated with the rate of change in the ALSFRS-R at 4 and 12 months (Fig. 3C,D). Thus, we observed a correlation between the MNC-NAIP level and ALS progression, indicating that the cumulative MNC-NAIP level led to the slow progression of ALS.

## Discussion

The cause and pathogenesis of ALS remain unclear, and many of the ALS drug candidates tested, excluding riluzole and edaravone, have not shown any apparent outcome in the past decade. The lack of a reliable biomarker in ALS is hampering the delineation of molecular pathogenesis and the development of therapeutics for ALS.

A previous ALS biomarker study assessed oxidative stress biomarkers, including coenzyme Q10, in clinical trials in ALS patients; however, negative results were obtained^30,31^. Other studies reported that creatinine and serum C-reactive protein could serve as biomarkers for survival^32–34^. Moreover, a recent study showed that the cerebrospinal fluid (CSF), serum, and plasma levels of neurofilament light chain (NfL)—a marker of axonal loss—were markedly higher in ALS patients as compared to the healthy controls. In spite of the relatively constant level of NfL level during follow-up period, NfL is a potential prognostic biomarker of ALS^35^.

In the present study, we focused on the endogenous antioxidative factor NAIP to explore a novel ALS biomarker, and accordingly examined the longitudinal change in the NAIP level in MNC from the peripheral blood of ALS patients. We observed that the MNC-NAIP level at baseline in ALS patients was lower than nearly half that of the healthy controls. In addition, ALS patients with a longitudinal and cumulative amount of the NAIP in MNC during the testing period showed a correlation with slow disease progression. Although the sample size is small, our study shows that the change in the MNC-NAIP level is directly or inversely associated with ALS progression.

Oxidative stress is thought to be one of the pathogenetic mechanisms for ALS^9^. Moreover, NAIP selectively suppresses oxidative stress-induced cell death *in vitro* and *in vivo*, and the overexpression of NAIP protects neuron and axonal regeneration^23–25^. Furthermore, a previous study demonstrated that treatment with NAIP-upregulating compound BRC sustains motoneuronal function in ALS patients^29^. NAIP upregulation protects motorneurons via suppression of microglial IL-1β generation and gliosis in ALS mice^23^. In the present study, we observed slow disease progression in ALS patients with cumulative/longitudinal increases in MNC-NAIP levels. Thus, NAIP might protect against motoneuronal dysfunction and neuronal inflammation via suppression of inflammatory factor production (including IL-1β) induced by oxidative stress; however, we have no evidence regarding the manner in which the NAIP level in MNC reflects that in glia cells and motoneuronal cells at this moment and it remains to be ruled out.

**Figure 4:**
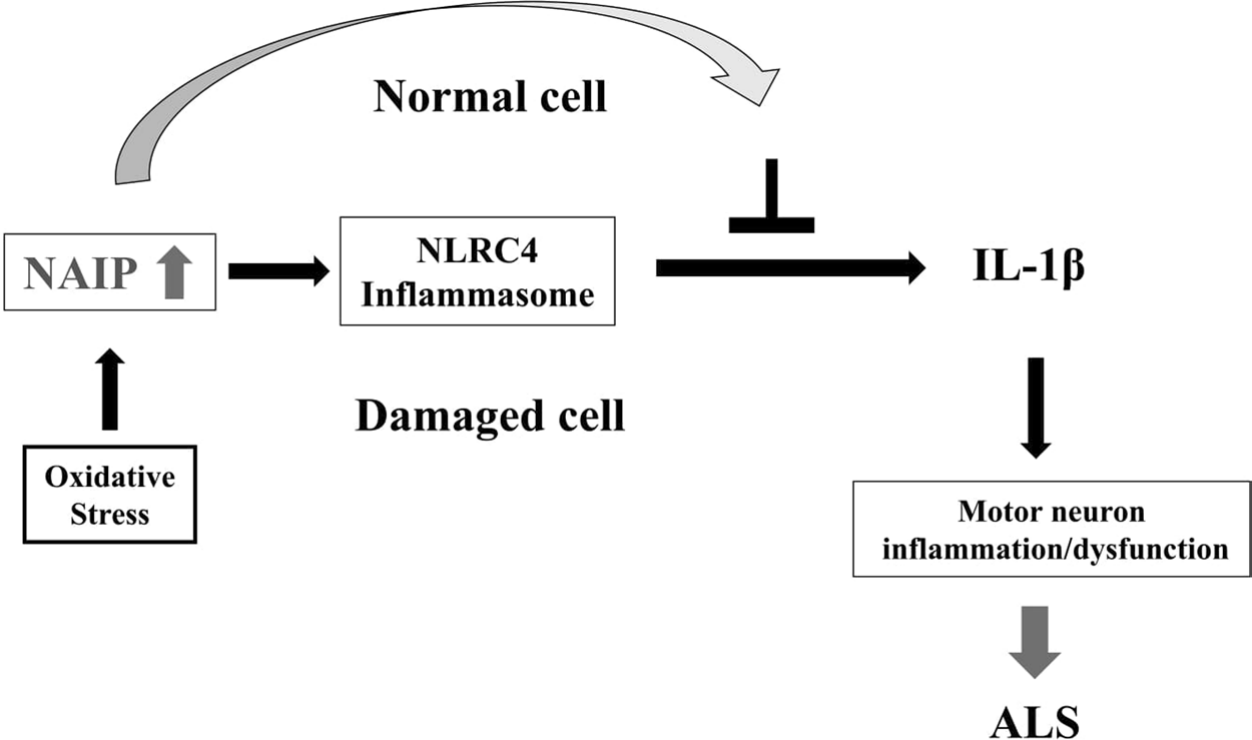
Model depicting the dual nature of NAIP. In normal neuronal cells: NAIP protects these cells against neuronal inflammation/dysfunction through the suppression of inflammatory factor production (including IL-1β) induced by oxidative stress. In damaged neuronal cells: NAIP commits the NLRC4 inflammasome complex, and induces IL-1β activation, resulting in induction of inflammation/dysfunction in motor neuron.

NAIP is a particularly well-characterized protein that is part of the NLR subfamily and constituents parts of NLRC4 inflammasome, and plays a role in innate immunity^36^. In ALS and inflammasome research, increased inflammasome activation appears to be involved in ALS^37^. Moreover, inflammasome activity is known to be regulated by reactive oxygen species (ROS)^38^. In the present study, we found that the NAIP level was approximately 50% lower in ALS patients than in healthy controls. Therefore, the reduction of NAIP at baseline in ALS patients may facilitate oxidative stress-induced damage, which may lead to ROS generation and consequently to the induction of inflammasome activation. These background events may lead to the chronic neuronal inflammation that triggers the onset of ALS. Thus, NAIP possesses an antinomic dual nature, which are protection of neuronal cells against oxidative stress on one hand and facilitation of inflammatory-induced cell death leading to apoptosis/necrosis to exclude damaged motoneuronal cells on the other hand (Fig. 4).

In the present study, we sought to address the following queries. First, we verified whether the change of the MNC-NAIP level was unique to ALS. To address this issue, we analyzed the MNC-NAIP levels in patients with Parkinson’s disease, and the NAIP level in MNC from Parkinson’s disease patients was as high as that of healthy controls, implying that the change of the MNC-NAIP level is not quite common to PD and could be neither for neurodegenerative disorders. Secondly, we considered from which stage the NAIP level was decreased in ALS patients. As shown in Table S1, it appears that there is no correlation between the disease duration of more or less than 2 years and the MNC-NAIP level at baseline. Additionally, weak correlation between the amount of MCN-NAIP and the change of ALSFRS-R score revealed at 4 months testing period (Fig. 3A; R^2^ = 0.1197, P = 0.29), but NAIP upregulation shown as the change of the rate of NAIP amount indicated a reliable commitment to slow ALS progression even at 4 months testing period (Fig. 3C; R^2^ = 0.30767, P = 0.07). Apparently, a correlation between the higher amount of NAIP and the slowing ALS progression became obvious at 12 months testing period (Fig. 3B; R^2^ = 0.79892, P = 0.016). Furthermore, as all the participants were taking riluzole, but not edaravone, during the entire course of our study, the treatment regimen was identical in all the ALS participants. Hence, we speculate that the effect of polypharmacy was extremely low. Therefore, the decrease in the NAIP level may initiate at the early stage of ALS or pre-symptomatic stage. Thirdly, we contemplated the mechanism underlying the longitudinal NAIP increase in the MNC. It has been reported that more inflammation related-gene expression are detected in monocytes in rapidly progressing rather than slower progressing ALS patients^39^. Considering that the previous studies suggested the link between inflammatory response and oxidative stress in ALS^23,40^ and NAIP expression was detected in MNC specifically (Supplementary Fig. S1), it is conceivable that MNC-NAIP is being upregulated by the signal released from the neuronal cell undergoing apoptosis/necrosis via NLRC4 inflammasome activation, and suppresses dominantly neuronal inflammation in healthy neural environment. Cumulative chronic inflammation breaches the healthy neural environment, and NLRC4 inflammasome activation turns to dominant to leading the neuronal degeneration and death.

On the other hand, the bulbar type in ALS is generally thought to result in rapid progression as compared to the limb type. In the present study, the ALSFRS-R score was decreased, particularly in the bulbar type, despite an increase in the MNC-NAIP level. This trend may be related to the severe neuronal inflammation which exceed the NAIP protection against motoneuronal dysfunction. Our present study also showed that a few healthy volunteer controls (C4-6) exhibited MNC-NAIP levels that were almost twice as high as the other controls. With regards to this difference levels, transient or persistent NAIP-upregulation may have occurred as a result of a hidden cancer or some other inflammatory causes.

In conclusion, to our knowledge, this is the first study to show that the MNC-NAIP level is low in ALS patients. Although the mechanism underlying the reduction of the NAIP level in peripheral blood MNC of ALS patients remains elusive, our data suggest that a lower NAIP level may be a risk factor for ALS. In addition, we observed a correlation between the cumulative expression of NAIP and the slowing of ALS progression, although in the small group analysis of the patients. These findings implying a role for NAIP as an ALS prognostic biomarker should be braced up by further large scale clinical study. Thus, our present study provides novel insights into the molecular pathogenesis of ALS. The development of strategies targeting the NAIP alteration in ALS patients may be useful as an evaluation and monitoring system for identifying novel ALS therapy.

## Methods

### Participants and sampling

This study was conducted between September 1, 2013, and December 31, 2015 on 18 Japanese ALS patients who met the revised El Escorial diagnostic criteria for definite, probable, and probable laboratory-supported ALS^41^. Healthy controls typically included spouses and unrelated caregivers. Clinical information was obtained at 4 month intervals up to 12 months. Symptom onset was defined as the first patient-reported weakness. The progression rate was calculated at baseline and at visits every 4 months using ALSFRS-R and forced vital capacity (%FVC). Taking of riluzole, induction of percutaneous endoscopic gastrostomy (PEG), and noninvasive ventilation (NIV) at the time of sampling was recorded. ALSFRS-R provides a physician-generated estimated of the patient’s degree of functional impairment, which can be evaluated serially to objectively assess any response to treatment or progression of disease. This score includes 12 questions that ask the physician to rate impression or the patient’s level of functional impairment (speech, salivation, swallowing, handwriting, cutting food, dressing and hygiene, turning in bed, walking, climbing stairs, dyspnea, orthopnea, respiratory insufficiency). Each task is rated on a five-point scale from 0 = can’t do, to 4 = normal ability. Individual item scores are summed to produce a reported score of between 0 = worst and 48 = best. This study was approved by the Ethical Committee of Toho University Omori Medical Center (ref. no. 2508125025, UMIN ID 000015449) and written informed consent was obtained from all of the participants prior to enrollment. This study was carried out in accordance with Ethical Guidelines for Medical and Health Research Involving Human Subjects by the Ministry of Education, Culture, Sports, Science and Technology.

### Antibody

With regard to the primary antibodies for Western blotting, anti-CD11b antibody (1:500, Assay Biotech), anti-CD16-B (Z64) mouse monoclonal IgG2b (1:200, Santa Cruz Biotechnology), anti-GAPDH (14C10) rabbit mAb (1:10000, Cell Signaling Technology), and anti-β-actin antibody (1:3000, Cell Signaling Technology) were used.

Anti-NAIP antiserum (1:5000, Neugen Pharma Inc.), which is commercially available (Wako, product code 019-24251), was raised by immunizing Japanese White rabbit with full-length NAIP synthesized using cell-free protein synthesis (WEPRO1240G Expression Kit) according to the manufacturer’s instruction^24^, and was used as the primary antibody for both Western blotting and dot blotting.

Anti-rabbit IgG, horseradish peroxidase (HRP)-linked whole antibody (1:10000, GE Healthcare) and anti-mouse IgG, HRP-linked whole antibody (1:10000, GE Healthcare) were used as secondary antibodies.

### Isolation of mononuclear cells from human peripheral blood

To measure the NAIP levels, peripheral blood MNC were isolated from human venous blood by density centrifugation. Five milliliters of the anticoagulated venous blood were carefully layered over 5 mL of Polymorphprep (Axis-Shield) in a 15-ml polypropylene conical tube. The tubes were centrifuged at 500 × *g* for >30 min using a swing-out rotor at room temperature. A Pasteur-pipette was inserted into the separated upper and lower bands, which consisted of MNC and polymorphonuclear cells, respectively; cells from each band were carefully harvested and suspended with an equal volume of 2-fold diluted PBS (Takara Bio Inc.). Thereafter, the cell suspension was mixed with an equal volume of PBS, and centrifuged at 400 × *g* for 10 min at room temperature. After discarding the supernatant, cells were resuspended with PBS and centrifuged at 400 × *g* for 10 min at room temperature; the supernatant was again discarded. When residual erythrocytes were detected in the collected-cell fraction, the contaminating erythrocytes were lysed by incubating the collected-cell fraction in 5 mL of ice-cold distilled water for 30 s, followed by the addition of an equal volume of 20 mM Hepes buffer (pH7.4) containing 1.8% (w/v) NaCl. After centrifugation at 400 × *g* for 10 min at room temperature, the supernatant was discarded. Cells were resuspended with 5 mL (MNC) or 4 mL (polymorphonuclear cells) of PBS, and the cell numbers were counted by Luna (LogosBiosystems) according to the manufacturer’s instruction. After centrifugation at 400 × *g* for 10 min at room temperature and discarding the supernatant, cells were mixed with 1 mL of PBS and transferred to a 1.5 mL tube. After another session of centrifugation at 400 × *g* for 10 min at room temperature and discarding the supernatant, the cells were stored at −80 °C until further use.

Approximately 100 μL of erythrocytes was suspended with an equal volume of 2-fold diluted PBS, and the cell suspension was mixed with an equal volume of PBS. After centrifugation at 400 × *g* for 10 min at room temperature and discarding the supernatant, the cells were stored at −80 °C until further use.

### Preparation of protein extract from MNC, polymorphonuclear cells, and erythrocytes

Frozen cells were suspended in M-PER mammalian protein extraction reagent (Thermo Fisher Scientific) supplemented with Protease Inhibitor Cocktail for Use with Mammalian Cell and Tissue Extracts (Nacalai Tesque), and lysed by incubation for 30 min on ice. Cell lysates were centrifuged at 14,000 × *g* for 15 min at 4 °C, and the supernatant was collected as protein extract. The protein concentration was determined by using a Pierce 660 nm Protein Assay kit (Thermo Fisher Scientific) and bovine serum albumin as a protein standard according to the manufacturer’s instructions.

### Western blotting

From the protein extract, 1 μg protein from MNC, 5 μg protein from polymorphonuclear cells, and 10 μg protein from erythrocytes were separated on 5–20% SDS-polyacrylamide gel (ATTO) and transferred onto a polyvinylidene difluoride (PVDF) membrane (Bio-Rad). Membranes were blocked with Blocking-One reagent (Nacalai Tesque) for 1 h at room temperature, and were incubated with the appropriate primary antibody in Can Get Signal solution 1 (TOYOBO) for 3–4 h at room temperature or overnight at 4 °C. Membranes were washed with TBST (20 mM Tris-HCl, pH 7.5, 150 mM NaCl, 0.1% [w/v] Tween20) 5 times for 5 min each, and then incubated with the HRP-conjugated secondary antibody in Can Get Signal solution 2 (TOYOBO) for 1 h at room temperature. After washing with TBST (5 times for 5 min each), signals were detected using Immobilon Western HRP substrate (Millipore) and were exposed on an X-ray film.

### Dot blotting

Proteins from healthy controls and ALS patients were diluted with PBS containing 0.001% (w/v) Ponceau S staining solution (Nacalai Tesque) to a concentration of 0.05 μg/μL. NAIP recombinant protein at 10 different concentrations (0–12.8 ng/20 μL) was prepared by diluting with PBS containing 0.001% (w/v) Ponceau S as the standard solution. Twenty microliters of the protein solutions and standard solutions were applied on the same PVDF membrane (11 × 7.5 cm) (Bio-Rad) set in a 96-well dot blot apparatus (DHM-96, Scie-Plas Ltd.), and were aspirated for 30 s using a vacuum pump in order to dot proteins on the PVDF membrane. The membrane was dried for 1 h at room temperature. Four sets of membranes were prepared, with the same protein amount on each membrane. After the PVDF membranes were rehydrated with methanol for 30 s and washed in TBST for 10 min, they were blocked with Blocking-One reagent (Nacalai Tesque) for 1 h at room temperature and then incubated with the anti-NAIP antiserum in Can Get Signal Solution 1 (TOYOBO) overnight at 4 °C. After washing with TBST 3 times for 10 min each, PVDF membranes were incubated with HRP-conjugated secondary anti-rabbit IgG (GE Healthcare) in Can Get Signal Solution 2 (TOYOBO) for 1 h at room temperature. After washing with TBST 3 times for 10 min each, the PVDF membranes were then treated with Immobilon Western HRP Substrate (Millipore) for 5 min, and the signals of each dot were visualized using an Amersham Imager 600 (GE Healthcare). The density of each dot was measured using ImageQuant TL analysis software (GE Healthcare). The amount of NAIP was calculated using a NAIP recombinant protein standard curve.

### Regression analysis

We performed regression analysis using JMP^®^ version 13.0.0 (SAS Institute Inc.). The parameter of the rate of change of ALSFRS-R was calculated using the following formula: (Initial ALSFRS-R score at baseline) − (ALSFRS-R score at 4 or 12 months)/(Initial ALSFRS-R score at baseline) × 100. The parameter of the rate of change of NAIP was calculated using the following formula: (Initial amount of NAIP at 4 or 12 months) − (Initial amount of NAIP)/(Initial amount of NAIP at baseline) × 100.

### Statistical analysis

Statistical analyses were performed with JMP^®^ version 13.0.0 (SAS Institute Inc.). Numerical variables are expressed as mean ± standard deviation (SD). We used 2-tailed t-tests and nonparametric Mann-Whitney U test or Pearson’s chi-square test for group comparisons, as appropriate. A P value < 0.05 was considered statistically significant.

## Electronic supplementary material

Supplementary Information
