# Supplementary material for: Neuronal apoptosis inhibitory protein is implicated in amyotrophic lateral sclerosis symptoms
Source: Sci Rep. 2018 Jan 8;8:6. doi: 10.1038/s41598-017-18627-w (PMC5758777; doi:10.1038/s41598-017-18627-w)
Supplement: Supplementary file 1 — Supplementary Information [file 41598_2017_18627_MOESM1_ESM.doc]

Supplementary information

Supplement to: Neuronal apoptosis inhibitory protein is implicated in amyotrophic lateral sclerosis symptoms

Osamu Kano, Kazunori Tanaka, Takuya Kanno, Yasuo Iwasaki, and Joh-E Ikeda, Ph.D.

Supplementary Appendix Table of Contents

Supplementary Appendix Part Ⅰ- Table S1

Supplementary Appendix Part Ⅱ - Figure S1

Supplementary Appendix Part Ⅲ - Figure S2

Supplementary Appendix Part Ⅰ - **Table S1. Characteristic of ALS and PD patients and normal controls.**

| **ALS** | **age, year** | **gender M/F** | **clinical onset** | **duration, month** | **NAIP,**  **ng/μg(SD)** |  |  |  | **ALSFRS-R** |  |  |  | **%FVC, %** |  |  |  | **riluzole** | **PEG** | **NIV** | **control** | **age, year** | **gender** | **NAIP, ng/μg(SD)** |
| --- | --- | --- | --- | --- | --- | --- | --- | --- | --- | --- | --- | --- | --- | --- | --- | --- | --- | --- | --- | --- | --- | --- | --- |
|  |  |  |  |  | baseline | 4 months | 8 months | 12 months | baseline | 4 months | 8 months | 12 months | baseline | 4 months | 8 months | 12 months |  |  |  |  |  |  |  |
| 1 | 60 | M | B | 42 | 0.50  (0.07) | 0.59  (0.04) | 0.48  (0.06) |  | 40 | 36 | 34 |  | 70.3 | 62.4 | 57.2 |  | Y | Y | N | 1 | 65 | F | 1.05  (0.20) |
| 2 | 66 | M | U | 13 | 0.49  (0.05) | 0.45  (0.07) |  |  | 37 | 33 |  |  | 72.9 | 40.8 |  |  | Y | Y | Y | 2 | 57 | F | 1.16  (0.06) |
| 3 | 61 | M | L | 33 | 0.53  (0.06) |  |  |  | 34 |  |  |  | 58.5 |  |  |  | Y | Y | Y | 3 | 71 | F | 1.07  (0.02) |
| 4 | 69 | F | U | 22 | 0.30  (0.01) |  |  |  | 32 |  |  |  | 40.9 |  |  |  | Y | Y | N | 4 | 42 | M | 2.35  (0.21) |
| 5 | 77 | M | U | 30 | 0.41  (0.05) |  |  |  | 32 |  |  |  | 66.6 |  |  |  | Y | Y | N | 5 | 77 | M | 2.27  (0.13) |
| 6 | 66 | M | B | 16 | 0.65  (0.04) | 0.72  (0.05) |  |  | 30 | 18 |  |  | 37.4 | 36.3 |  |  | Y | Y | N | 6 | 70 | F | 2.22  (0.31) |
| 7 | 58 | M | U | 16 | 0.55  (0.02) | 0.90  (0.07) | 0.93  (0.05) | 0.62  (0.06) | 47 | 43 | 37 | 33 | 62.2 | 42.5 | 36.7 | 34.8 | Y | N | N | 7 | 54 | F | 1.23  (0.10) |
| 8 | 71 | F | B | 18 | 0.28  (0.03) | 0.74  (0.06) | 0.87  (0.09) | 0.58  (0.14) | 40 | 38 | 34 | 30 | 85.8 | 54.9 | 50.7 | 40 | Y | Y | Y | 8 | 72 | F | 0.73  (0.12) |
| 9 | 58 | M | U | 77 | 0.75  (0.02) | 0.89  (0.07) | 1.39  (0.21) | 1.78  (0.35) | 34 | 34 | 34 | 34 | 97.1 | 103.2 | 87.2 | 89.1 | Y | N | N | 9 | 64 | F | 0.66  (0.08) |
| 10 | 30 | M | L | 16 | 0.63  (0.11) | 0.71  (0.09) | 0.69  (0.07) | 1.12  (0.04) | 46 | 44 | 43 | 41 | 84.1 | 82 | 76.6 | 64.8 | Y | Y | Y | 10 | 56 | F | 0.83  (0.22) |
| 11 | 65 | M | U | 23 | 0.68  (0.09) | 1.58  (0.09) | 0.70  (0.08) | 1.19  (0.10) | 45 | 45 | 43 | 42 | 107.3 | 104.6 | 103 | 91.8 | Y | N | N | 11 | 45 | M | 0.98  (0.23) |
| 12 | 44 | F | U | 38 | 0.31  (0.02) | 0.70  (0.04) | 0.56  (0.05) | 0.71  (0.10) | 30 | 29 | 29 | 26 | 107.4 | 103.3 | 99.3 | 96.3 | Y | Y | Y | 12 | 44 | M | 1.49  (0.29) |
| 13 | 78 | M | B | 34 | 1.07  (0.02) | 0.72  (0.05) |  |  | 19 | 13 |  |  | 31.4 | 26.8 |  |  | Y | N | N |  |  |  |  |
| 14 | 79 | F | B | 28 | 0.59  (0.09) |  |  |  | 39 |  |  |  | 53.1 |  |  |  | Y | Y | Y |  |  |  |  |
| 15 | 68 | F | L | 10 | 0.50  (0.07) | 0.54  (0.06) |  |  | 44 | 36 |  |  | 134.1 | fatigue |  |  | Y | N | N |  |  |  |  |
| 16 | 78 | M | U | 18 | 0.96  (0.12) |  |  |  | 32 |  |  |  | 96.5 |  |  |  | Y | N | N |  |  |  |  |
| 17 | 67 | M | U | 47 | 0.52  (0.11) |  |  |  | 46 |  |  |  | 100.9 |  |  |  | Y | N | N |  |  |  |  |
| 18 | 58 | M | B | 16 | 1.43  (0.19) |  |  |  | 40 |  |  |  | 85 |  |  |  | Y | Y | Y |  |  |  |  |

| **PD** | **age, year** | **gender M/F** | **H&Y scale** | **duration, month** | **NAIP,**  **ng/μg(SD)** | **LEDD**  **(mg)** | **dopamine**  **agonist** |
| --- | --- | --- | --- | --- | --- | --- | --- |
| 1 | 75 | M | 2 | 57 | 0.36  (0.05) | 880 | Y |
| 2 | 66 | M | 3 | 50 | 0.70  (0.19) | 600 | Y |
| 3 | 72 | M | 3 | 15 | 0.34  (0.03) | 300 | N |
| 4 | 70 | F | 3 | 107 | 0.82  (0.03) | 463 | Y |
| 5 | 69 | M | 2 | 33 | 0.58  (0.05) | 395 | Y |
| 6 | 73 | F | 3 | 140 | 0.77  (0.11) | 1023 | N |
| 7 | 71 | F | 3 | 84 | 1.39  (0.05) | 600 | N |
| 8 | 69 | M | 2 | 63 | 1.38  (0.13) | 495 | Y |
| 9 | 67 | F | 3 | 79 | 0.73  (0.14) | 575 | Y |
| 10 | 72 | M | 4 | 68 | 1.81  (0.13) | 757 | Y |
| 11 | 72 | M | 3 | 158 | 1.12  (0.05) | 525 | Y |

SD: standard deviation, M: male, F: female, M: men, F: female, B: bulbar type, U: upper limb type, L: lower limb type, NAIP: neuronal apoptosis inhibitory protein, ALSFRS-R: Amyotrophic Lateral Sclerosis Functional Rating Scale-Revised, PEG: percutaneous endoscopic gastrostomy, NIV: noninvasive ventilation, H&Y scale: Hoehn and Yahr scale, LEDD: Levodopa equivalent daily dose, Y: yes, N: no

Supplementary Appendix Part Ⅱ - **Figure S1. NAIP expresses superiority in peripheral blood mononuclear cells from healthy controls and ALS patients.**

The expression of NAIP in mononuclear cells (A), polymorphonuclear cells (B), and erythrocytes (C) from 3 healthy controls (lanes 1-3) and 3 ALS patients (lanes 4-6) was analyzed. The extracts from mononuclear cells (1 μg protein), polymorphonuclear cells (5 μg proteins) and erythrocytes (10 μg proteins) were used for Western blotting with anti-NAIP antiserum (upper panels). CD11b and CD16b were used as mononuclear cell and polymorphonuclear cell markers, respectively (middle panels). Glyceraldehyde-3-phosphate dehydrogenase (GAPDH) and β-actin were used as internal controls for the extracts from mononuclear cells and erythrocytes and for the extract from polymorphonuclear cells, respectively (bottom panels). Closed arrowhead shows the position of NAIP signal. Asterisks denote non-specific signal. The expression of NAIP was not detected in polymorphonuclear cells and erythrocytes by the long exposure. The thin signals specifically detected on the gel of the mononuclear fraction (A) were dimeric- and cleaved-peptide of NAIP.


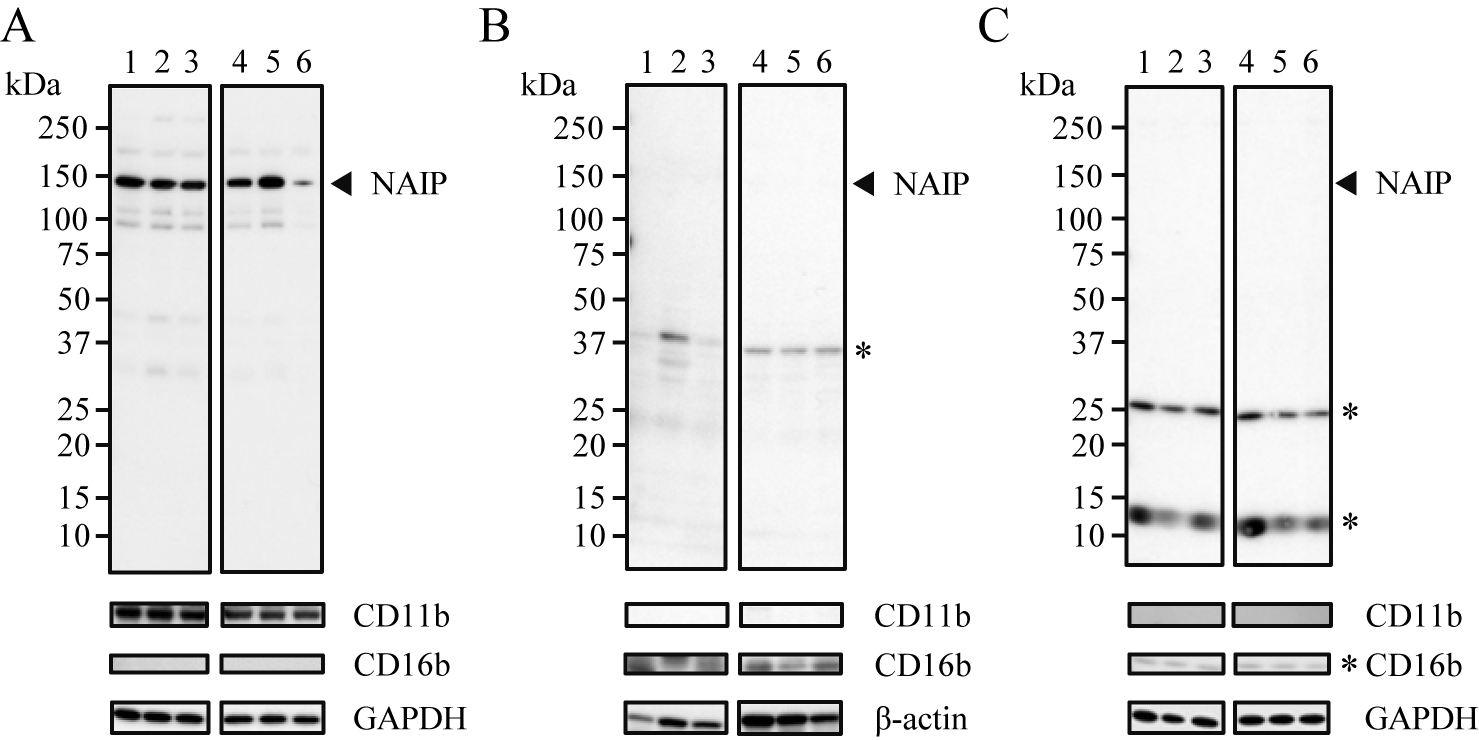


Supplementary Appendix Part Ⅲ - **Figure S2. Uncropped images for western blots.**

**
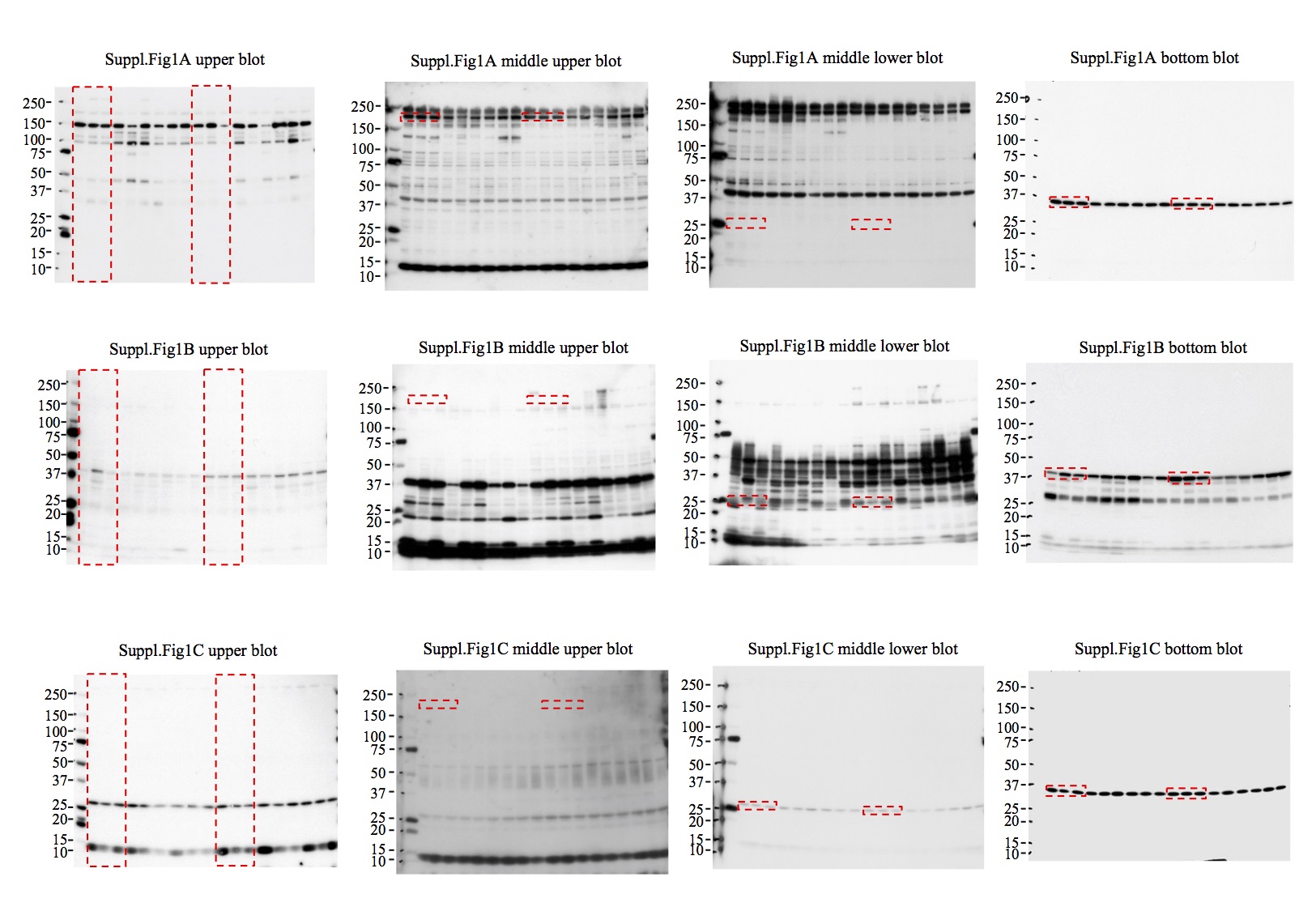
**
